# Beneficial effects of metformin on energy metabolism and visceral fat volume through a possible mechanism of fatty acid oxidation in human subjects and rats

**DOI:** 10.1371/journal.pone.0171293

**Published:** 2017-02-03

**Authors:** Ichiro Tokubuchi, Yuji Tajiri, Shimpei Iwata, Kento Hara, Nobuhiko Wada, Toshihiko Hashinaga, Hitomi Nakayama, Hiroharu Mifune, Kentaro Yamada

**Affiliations:** 1 Division of Endocrinology and Metabolism, Department of Internal Medicine, Kurume University School of Medicine, Kurume, Japan; 2 Institute of Animal Experimentation, Kurume University School of Medicine, Kurume, Japan; Stellenbosch University, SOUTH AFRICA

## Abstract

**Objective:**

Metformin is known to have a beneficial effect on body weight and body composition, although the precise mechanism has not been elucidated yet. The aim of this study is to investigate the effects of metformin on energy metabolism and anthropometric factors in both human subjects and rats.

**Methods:**

In human studies, metformin (1500mg/day) was administered to 23 healthy subjects and 18 patients with type 2 diabetes for 2 weeks. Metabolic parameters and energy metabolism were measured during a meal tolerance test in the morning before and after the treatment of metformin. In animal studies, 13 weeks old SD rats were fed 25–26 g of standard chow only during 12-hours dark phase with either treated by metformin (2.5mg/ml in drinking water) or not for 2 weeks, and metabolic parameters, anthropometric factors and energy metabolism together with expressions related to fat oxidation and adaptive thermogenesis were measured either in fasting or post-prandial state at 15 weeks old.

**Results:**

Post-prandial plasma lactate concentration was significantly increased after the metformin treatment in both healthy subjects and diabetic patients. Although energy expenditure (EE) did not change, baseline respiratory quotient (RQ) was significantly decreased and post-prandial RQ was significantly increased vice versa following the metformin treatment in both groups. By the administration of metformin to SD rats for 2 weeks, plasma levels of lactate and pyruvate were significantly increased in both fasting and post-prandial states. RQ during a fasting state was significantly decreased in metformin-treated rats compared to controls with no effect on EE. Metformin treatment brought about a significant reduction of visceral fat mass compared to controls accompanied by an up-regulation of fat oxidation-related enzyme in the liver, UCP-1 in the brown adipose tissue and UCP-3 in the skeletal muscle.

**Conclusion:**

From the results obtained, beneficial effects of metformin on visceral fat reduction has been demonstrated probably through a mechanism for a potential shift of fuel resource into fat oxidation and an upregulation of adaptive thermogenesis independent of an anorexigenic effect of this drug.

## Introduction

In the 2013 issue of the International Diabetes Federation (IDF) Diabetes Atlas, the prevalence of diabetes in the Western Pacific (WP) Region was reported to be 8.6% in 2013, or 138 million adults, and was estimated to rise to 11.1%, or 201 million adults, in 2035 [1]. Type 2 diabetes accounts for 95% of the diabetes in Japanese diabetic patients and the number of patients is still increasing along with the number of overweight/obesity individuals reflecting environmental factors, including overeating and a lack of exercise. However, it is of interest that the prevalence of diabetes in Asian people is almost the same as that in Caucasian despite of their much lower BMI. The reason is probably genetic and is likely due to their lower capacity for insulin secretion in comparison to Caucasian’s [2]. Asian people, including Japanese, are therefore thought be much more likely to be affected by diabetes than people in western countries in the era of satiety.

Generally, most glucose-lowering strategies including the administration of oral hypoglycemic agents (OHAs) such as sulfonylurea and thiazolidinedione and insulin injections for the treatment of diabetes are associated with the potential risk of weight gain, and may lead to a worsening of blood glucose control [3, 4]. In contrast, metformin is known to have a neutral effect on body weight and was shown to reduce the amount of body fat and improve body composition in previous studies performed in type 2 diabetic patients [5, 6]. These beneficial effects of metformin on body weight and composition have mostly been discussed in relation to its anorexigenic effect, which has thus far been demonstrated in mice [7] and rats [8] so far. The anorexigenic effect of metformin, along with weight reduction and the improvement of blood pressure, lipid profile and glucose control has also been reported in patients with type 2 diabetes [9] and other life style-related disease [10]. It is well established that metformin primarily lowers blood glucose concentrations by suppressing hepatic glucose production [11] and ameliorating hepatic insulin resistance. Metformin leads to the accumulation of AMP in the liver, resulting in the inhibition of adenylate cyclase [12]. The reduction of cAMP levels and protein kinase A activity suppresses glucagon-induced gluconeogenesis in the liver.

Metformin has also been demonstrated to increase AMP-activated protein kinase (AMPK) activity in rat hepatocytes and in rat and human skeletal muscle *in vitro* [13, 14]. AMPK is a serine-threonine kinase that responds to fluctuations in cellular energy levels and which is activated in situations of energy consumption, thus it has the function of maintaining energy homeostasis [15, 16]. In skeletal muscle, AMPK is activated during exercise and is involved in contraction-stimulated glucose transport and fatty acid oxidation. In the liver, AMPK inhibits the production of glucose, cholesterol and triglyceride and stimulates fatty acid oxidation. AMPK regulates several of the key proteins involved in lipid metabolism. AMPK phosphorylates and inactivates acetyl-Co-A carboxylase (ACC), a rate-limiting enzyme for fatty acid synthesis [17]. The inactivation of ACC by AMPK in the liver results in a decrease in malonyl-CoA, higher carnitine palmitoyl transferase 1 (CPT-1) activity and the enhancement of fatty acid oxidation [18]. Furthermore, it has been reported that metformin enhanced expressions of uncoupling proteins (UCPs), which are key enzymes of adaptive thermogenesis, both in animal study [19] and in vitro culture study [20]. Thus, it is hypothesized that the administration of metformin promotes the reduction of body fat amount via the acceleration of fat oxidation and adaptive thermogenesis *in vivo*. However, little is known about the effects of metformin on fat oxidation, which not only include the effects on energy metabolism that are directly measured by respiratory gas analysis but also the precise molecules that are related to fat oxidation and thermogenesis, and consequently to its beneficial effects on body composition *in vivo*. The present study was performed to clarify these issues by simultaneously investigating metformin’s effects on energy metabolism and anthropometric factors in both human subjects and animals.

## Methods

### Human studies

#### Subjects

23 healthy volunteers (16 males, 28 ± 3 years old, BMI 22.0 ± 4.1 kg/m^2^) and 18 drug-naïve patients with type2 diabetes mellitus (10 males, 42 ± 16 years old, BMI 31.2 ± 6.7 kg/m^2^, HbA1c 9.1 ± 2.1%) were enrolled in the present study. Healthy subjects were recruited by advertisement, and diabetic subjects were recruited randomly from patients who had been admitted to the Division of endocrinology and Metabolism, Kurume University Hospital from March 2013 to October 2014. All participants provided written informed consent. All procedures were compliant with the Declaration of Helsinki, and the experimental protocol was approved by the ethical committee of Kurume-University (study number: 10152).

#### Experimental protocol

Metformin hydrochloride (1500 mg/day) was administered either 23 healthy subjects or 18 type 2 diabetic patients for 2 weeks. Subjects started taking metformin from small dose with gradual increasing (250mg twice daily on 1^st^ and 2^nd^ day, 500mg twice daily on 3^rd^ and 4^th^ day, 500mg three times daily on 5^th^ day and thereafter). After an overnight fast, meal tolerance tests (592 kcal, 75 g of carbohydrate, 28.5 g of fat; Saraya Co., Osaka, Japan) were performed in the morning twice before and after the 2 week-administration of metformin. On the meal tolerance test, blood samples were collected and respiratory gas analysis was performed before and 1, 2, 3 hours after an ingestion of meal. Energy intake was pre-specified to each diabetic patient base on their height and body weight, and the amount of actual food intake was checked at every meal for 2 weeks of metformin treatment.

#### Measurements

Plasma glucose, serum triglyceride levels were assessed according to the standard procedures. Plasma concentrations of insulin were measured with standard ELISAs. Blood levels of lactate were determined enzymatically with spectrophotometric assays. The volume of oxygen consumed (VO_2_) and the volume of carbon dioxide produced (VCO_2_) were measured with an indirect calorimetry (Oxycon Alpha, Fukuda-Denshi, Tokyo, Japan) for the calculation of energy expenditure (EE) and respiratory quotient (RQ) according to the formula as below [21].

$$EE\;\left(\frac{kcal}{min}\right)=3.815\times VO_{2}+1.232\times VCO_{2}$$

$$RQ=VCO_{2}÷VO_{2}$$

After a period for 15 min at rest, respiratory gas analysis was performed for 10 or 15 min at each time point.

### Animal studies

#### Animals

Male Sprague-Dawley (SD) rats (Jcl:SD, CREA Co Ltd, Osaka, Japan) were used in this study. The animals were housed in a controlled room (temperature 25 ± 2°C, humidity 60 ± 10%) under a 12 h light-dark cycle (light on 7:00–19:00). All the experiments were performed in accordance with protocols approved by the Kurume University Animal Experiment Committee, based on the NIH Guidelines for the Care and Use of Laboratory Animals (NIH publication, 1996). All surgical procedures were performed under 3% isoflurane (Wako Pure Chemical Industries, Ltd.,Osaka, Japan), and all efforts were made to minimize suffering.

#### Experimental protocol

A preliminary experiment was performed to measure food consumption of 11-week-old SD rats during light phase and dark phase, respectively. Rats fed 24.2±0.7g of standard chow (10 kcal% fat, produced by Research Diets, Inc., New Brunswick, NJ, USA: 23 open source diet code D12450B) per whole day, and more than 90% was consumed during dark phase. Thus we determined 25-26g of feeding only during dark period to clearly differentiate fasting and post-prandial state in the following experiments.

Rats at 12 weeks of age (n = 24) were started to be fed only during 12-h dark period for the acclimation to fasting and feeding rhythm for one week as mentioned above. 25–26 g of pellet chow was fed at 19:00 and withdrawn at 7:00 after the measurement of food intake. At 13 weeks old, rats were divided into two groups (n = 12 in each group); given drinking water either containing metformin hydrochloride (2.5 mg/ml) or not. All rats were housed individually in clear plastic TPX® cages (W27 × D43 × H20 cm) with paper bedding. Body weight was measured every week from 13 to 15 weeks of age. Food intake during dark period and amount of drinking for a whole day were measured at 7:00 every day.

#### Analysis of respiratory gas and body composition

After 2 weeks, rats at 15 weeks old were moved individually into acrylic metabolic chambers equipped with gas analysis system (ARCO system, Chiba) for two days for the measurement of oxygen consumption and respiratory quotient. The system consists of eight acrylic metabolic chambers, a mass spectrometer (model ARCO-2000) and a gas sampler (model ARCO-2000-GS10). Each metabolic chamber had a room (752 cm^2^ floor and 20 cm in height), and room air was pumped through the chambers at a rate of 2.0 L/min. The air from each chamber was sampled for 15 seconds. During the last 5 seconds, VO_2_ and VCO_2_ concentration were measured, and EE and RQ were calculated as mentioned in human study. The respiratory data for each chamber were obtained every 5 min, and the mean and cumulative data for 12 h during light period or 24 h were calculated from 144 or 288 samplings, respectively.

After the measurement of respiratory gas, visceral and subcutaneous fat volumes (mm^3^) were measured using in vivo micro-computed tomography (R_mCT2, Rigaku Co., Tokyo, Japan) under imaging conditions of FOV73 (φ73 mm×H57 mm), 90 kV tube voltage and 160 μA tube current. Rats were anesthetized with 3% isoflurane and placed supine in the machine, and serial 4 mm scans were performed from the anterior to the posterior aspect of 4^th^ lumbar vertebra. Fat analysis software (Rigaku Co., 24 Tokyo, Japan) estimated the volumes of adipose tissue, bone, air and the remainder on the basis of their different x-ray densities, and distinguished visceral and subcutaneous fat tissues by detecting the abdominal muscle layers.

#### Sampling and blood measurement

After the completion of anthropometric measurement, rats were sacrificed under anesthesia using 3% isoflurane in either fasting (19:00) or post-prandial (7:00) state, 6 rats in each time point. Blood samples were collected via abdominal aorta, and treated immediately as described below. Liver, brown adipose tissue (BAT) and skeletal muscle were obtained and cut into small pieces and immediately frozen in liquid nitrogen, then stored at −80°C.

Blood glucose concentrations were measured with a handheld glucose meter (One Touch Ultra; LifeScan, Milpitas, CA) immediately after blood sampling. Blood lactate and pyruvate were determined enzymatically with spectrophotometric assays. The remaining blood samples were centrifuged (3000rpm, 10min), and serum samples were separated and stored at −80°C until the assay. Serum concentrations of insulin were determined with a Rat Insulin ELISA KIT (Shibayagi, Gunma, Japan).

#### Quantitative real-time RT-PCR

Expressions of fat oxidation-related enzyme such as Acyl-CoA synthase, CPT-1, Acyl-CoA dehydrogenase, pyruvate dehydrogenase kinase (PDK) and adaptive thermogenesis-related molecules such as UCP-1, UCP-3 were measured by quantitative real time-PCR as described previously [22]. RNA was isolated using RNA-Bee (Cosmo Bio, Tokyo, Japan), and 5 μg of total RNA was reverse-transcribed to cDNA using a kit from Invitrogen (Carlsbad, CA, USA). SYBR green-based real-time quantitative PCR of cDNA templates was performed using StepOnePlus (Applied Biosystems, Foster City, CA, USA). The PCR cycling conditions were 10 min at 95°C followed by 40 cycles of 30 sec at 95°C, 30 sec at 53–64°C, and 30 sec at 72°C. The results were calculated as the expression of the target gene relative to the expression of the glyceraldehyde-3-phosphate dehydrogenase (*Gapdh*) gene. Forward and reverse primer sequences used in this study are shown in S1 Table.

#### Western blot analysis

Protein levels of AMPKα, phosphorylated AMPKα (pAMPKα), ACC, phosphorylated ACC (pACC) from fasting samples were measured by Western blot analysis as described previously [20]. Liver tissue was lysed in ice-cold lysis buffer containing 1 mmol/l dithiothreitol (DTT), 0.0025% NP40 and a cocktail of proteinase inhibitors. The lysate was centrifuged at 19,000 g for 15 min at 4°C, and the supernatant was collected as whole-cell extract. The total protein concentration of the whole-cell was measured using the Bradford reagent (Bio Rad, Hercules, CA, USA). After being heated at 100°C for 5 min, 20 μg total protein was loaded into each well, separated by 7.5% SDS-PAGE (Wako, Osaka, Japan) and transferred to a nitrocellulose membrane. The membrane was incubated with rabbit polyclonal antibodies against AMPKα, pAMPKα (Thr172), ACC, pACC (Ser79) or rabbit monoclonal antibody against GAPDH (Cell Signaling Technology, Danvers, MA, USA) at 4°C overnight. After being washed, the membrane was incubated with peroxidase-conjugated goat anti-rabbit IgG (Wako) and then visualized using an ECL system (GE Healthcare, Buckinghamshire, UK).

#### Pyruvate tolerance test

In another series of experiment with the same protocol, intraperitoneal pyruvate tolerance test was performed in SD rats at 15 weeks old with or without metformin treatment for 2 weeks (n = 6 in each group). After 12 h of fasting, a sodium pyruvate solution (250 mg/ml) was injected ip at a dosage of 2 g/kg. Glucose levels were determined in blood extracted from the tail before (0 min) and 15, 30, 60, 90 and120 min after an ip pyruvate injection.

### Statistical analysis

All tests were performed using JMP Pro Ver. 11 (SAS Institute Inc., USA). In human studies, data are presented as the means ± S.D. Statistical significance was determined by paired t-test. In animal studies, data are presented as the means ± S.E. Statistical significance was determined by unpaired Student’s t-test. A p-value < 0.05 was considered to be statistically significant.

## Results

### Human studies

Although 2 healthy subjects and 2 diabetic patients complained bloating and 3 healthy subjects and 2 diabetic patients complained mild diarrhea after the administration of metformin, no one was obliged to quit the medication due to adverse effects. Obvious anorexigenic effect was not observed in either healthy subjects or diabetic patients during 2-week metformin treatment.

**Fig 1:**
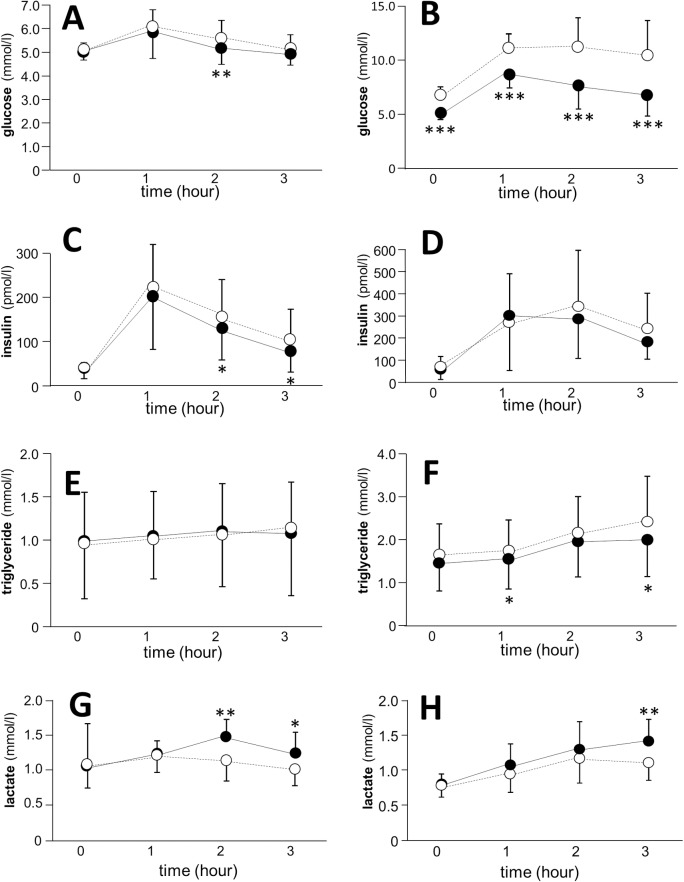
**Effects of metformin on metabolic factors in healthy subjects (A, C, E, G) and diabetic patients (B, D, F, H).** A, B; glucose, C, D; insulin, E, F; triglyceride, G, H; lactate. Data are presented as mean ± S.D. Paired t test was used for the comparison between groups. Open circle and dotted line; before metformin treatment, closed circle and solid line; after a 2-week treatment by metformin. *P<0.05, **P<0.01, ***P<0.001 vs. the value before metformin treatment.

Significant decreases of plasma glucose concentrations were observed at all points in patients with type 2 diabetes and 2 hours after meal in healthy subjects compared to values before administration (Fig 1). Although BMI in healthy subjects did not change significantly (22.4 ± 4.1 to 22.0 ± 4.1), a significant decrease of BMI was observed in diabetic patients by the administration of metformin (31.2 ± 6.7 to 30.6 ± 6.6, P<0.0001). The administration of metformin brought about significant reductions of serum insulin levels after 2 and 3 hours in healthy subjects and serum triglyceride concentrations after 1 and 3 hours of cookie ingestion in diabetic patients. Blood lactate levels after cookie ingestion were significantly increased by the administration of metformin in both the healthy subjects and diabetic patients (Fig 1).

**Fig 2:**
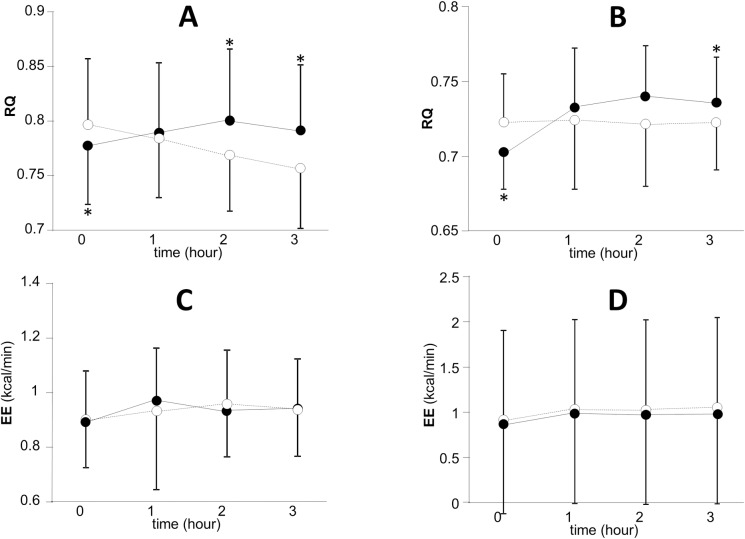
**Effects of metformin on respiratory quotient (RQ) and energy expenditure (EE) in healthy subjects (A and C) and diabetic patients (B and D).** A, B; RQ, C, D; EE. Data are presented as mean ± S.D. Paired t-test was used for the comparison between groups. Open circle and dotted line; before metformin treatment, closed circle and solid line; after a 2-week treatment by metformin. *P<0.05 vs. the value before metformin treatment.

Although there was no difference in EE, a significant decline of RQ before meal was brought about by the administration of metformin in both healthy subjects and diabetic patients. Conversely, metformin administration significantly enhanced post-prandial RQ in both healthy subjects and diabetic patients (Fig 2).

### Animal studies

Body weight, food intake, and water consumption did not differ between the two groups at any point for two weeks of metformin treatment. However, visceral fat volume was significantly decreased by the treatment of metformin while subcutaneous fat did not differ significantly between the groups (Table 1).

**Table 1:** Anthropometric factors, food intake and water consumption in control and metformin-treated rats.

	treatment	13 weeks	14 weeks	15 weeks	
**body weight (g)**	control	413 ± 9	451 ± 6	462 ± 5	
	metformin	414 ± 9	443 ± 6	453 ± 6	
**subcutaneous fat volume (cm^3^)**	control			2.44 ± 0.12	
	metformin			2.37 ± 0.13	
**visceral fat volume (cm^3^)**	control			8.46 ± 0.39	
	metformin			7.02 ± 0.41**	
**food intake (g/day)**	control	24.8 ± 0.2	25.2 ± 0.4	25.0 ± 0.3	
	metformin	24.3 ± 0.5	25.2 ± 0.4	24.8 ± 0.4	
**water consumption (ml/day)**	control	51.1 ± 3.1	63.3 ± 2.8	49.1 ± 2.5	
	metformin	49.7 ± 5.4	57.4 ± 3.3	43.4 ± 1.8	

Data are presented as mean ± S.E. n = 12 in each group.

**P<0.01 vs. control group.

**Fig 3:**
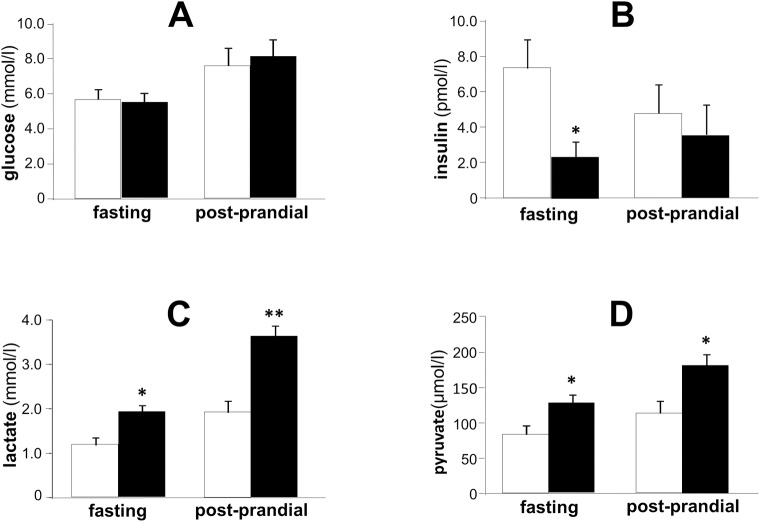
**Effects of metformin on plasma concentrations of glucose (A), insulin (B), lactate (C) and pyruvate (D) in SD rats at 15 weeks old.** Open bars denote control group and black bars denote metformin-treated group. Data are presented as mean ± S.E. (n = 6 in each group). *P<0.05, **P<0.01 vs. control group.

Although plasma glucose levels were almost the same between control and metformin-treated rats during fasting and post-prandial state, fasting plasma insulin levels were significantly lower in metformin-treated rats than control rats. Moreover, plasma concentrations of lactate and pyruvate were significantly (P<0.05) increased in both fasting and post-prandial state by the treatment of metformin for 2 weeks (Fig 3).

**Fig 4:**
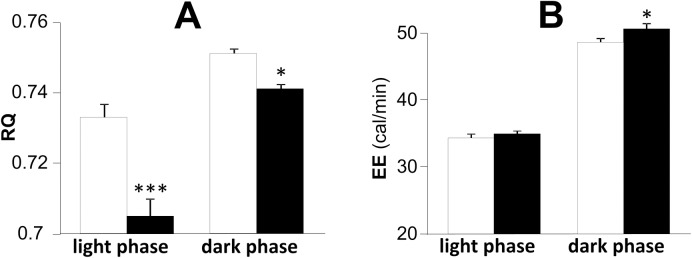
**Effects of metformin on respiratory quotient (RQ, A) and energy expenditure (EE, B) in SD rats at 15 weeks old.** Open bars denote control group and black bars denote metformin-treated group. Data are presented as mean ± S.E. (n = 10 in each group). *P<0.05, ***p<0.001 vs. control group.

Although EE did not change during light phase, slight but significant increase of EE was observed during dark phase by the administration of metformin. RQ during light phase decreased significantly in metformin-treated rats compared to control rats. There was slight but significant decrease in RQ during post-prandial state by metformin treatment (Fig 4).

**Fig 5:**
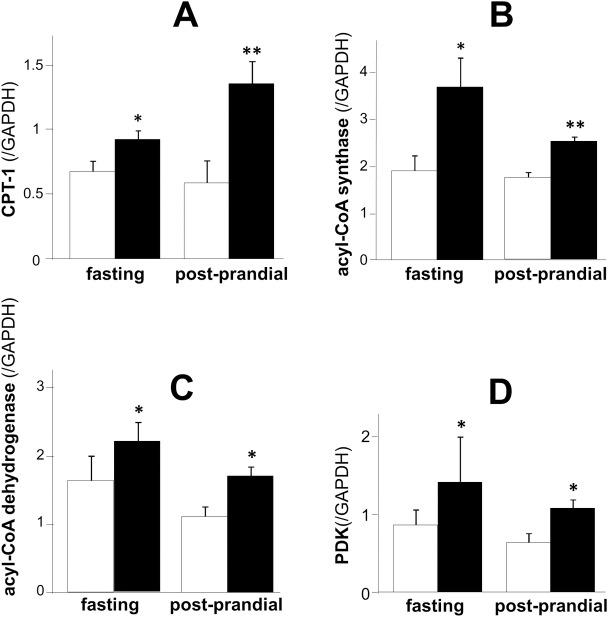
Fat oxidation-related gene expressions in the liver of SD rats at 15 weeks old. A; *Cpt1*, B; *Acs*, C; *Acad*, D; *Pdk* expression corrected by *Gapdh* expression. Open bars denote control group and black bars denote metformin-treated group. Data are presented as mean ± S.E. (n = 6 in each group). *P<0.05, **P<0.01 vs. control groups.

Expression of fat oxidation related enzymes such as *Pdk*, *Cpt1*, *Acs*, and *Acad* were significantly higher in metformin-treated rats than those in control rats during both fasting and post-prandial states. (Fig 5)

**Fig 6:**
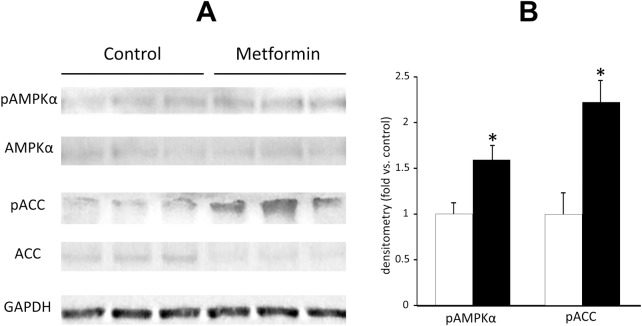
**The protein expressions of pAMPK and pACC in the liver of SD rats at 15 weeks old.inhibo** A; Western blot analysis, B; Densitometric quantification. Open bars denote control group and black bars denote metformin-treated group. Data are presented as mean ± S.E. (n = 3 in each group). *P<0.05 vs. control groups.

As shown in Fig 6, by the treatment of metformin the protein levels of phosphorylated AMPK and ACC, key molecules of fat oxidation, were significantly enhanced in the liver during fasting state.

**Fig 7:**
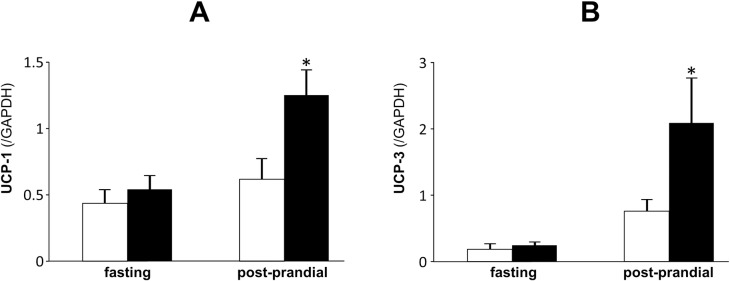
Adaptive thermogenesis-related gene expressions in the brown adipose tissue and skeletal muscle of SD rats at 15 weeks old. A; *UCP1*, B; *UCP3* expression corrected by *Gapdh* expression. Open bars denote control group and black bars denote metformin-treated group. Data are presented as mean ± S.E. (n = 6 in each group). *P<0.05 vs. control groups.

Results of gene expressions related to adaptive thermogenesis are described in Fig 7. During post-prandial state, UCP-1 in the BAT and UCP-3 in the skeletal muscle were markedly enhanced by the administration of metformin in spite of no effects of this drug during fasting state.

**Fig 8:**
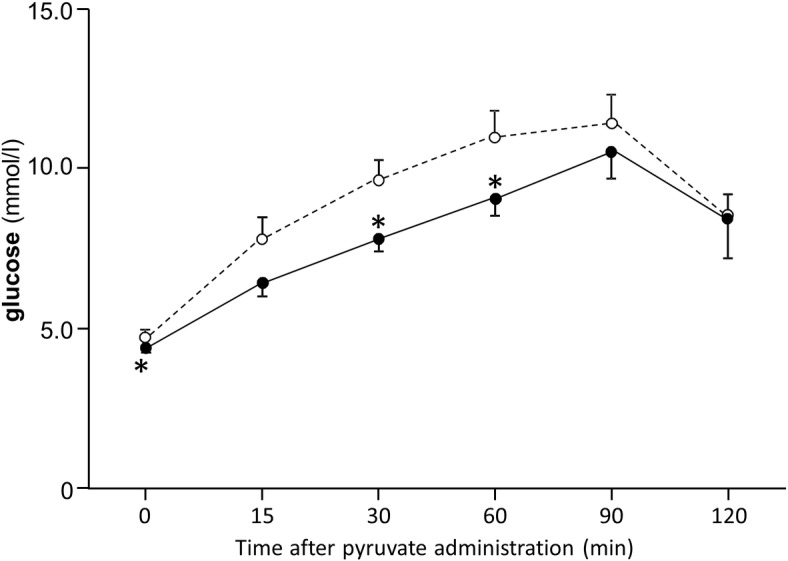
Pyruvate tolerance test in SD rats at 15 weeks old. Data are presented as mean ± S.E. (n = 6 in each group). Paired t-test was used for the comparison between groups. Open circle and dotted line; control group, closed circle and solid line; metformin-treated group. *P<0.05 vs. the value of control group at each time point.

In pyruvate tolerance test, metformin treatment brought about a significant reduction of blood glucose levels before and 30, 60 minutes after the intraperitoneal administration of pyruvate (Fig 8).

## Discussion

The principal results of the present study are as follows. First, lactate and pyruvate concentrations increased, reflecting the enhancement of anaerobic glycolysis by metformin treatment [23]. Second, metformin decreased RQ, suggesting that fat oxidation was accelerated by this drug. Third, metformin reduced visceral fat in rats, independent of its anorexigenic effect. Finally, metformin was found to upregulate enzymes related to fat oxidation and adaptive thermogenesis, in line with a lower RQ and visceral fat mass reduction.

Metformin is known to have a neutral effect on body weight or, based on the results of a meta-analysis, to possibly decrease it by 1.1kg [24]. Metformin’s effects on body weight have thus far mostly been discussed in relation to its anorexigenic effect in animal models [7, 8] and human subjects [9, 10]. Indeed, metformin has been reported to reduce meal size without altering the number of meals in obese db/db mice, suggesting that metformin modifies satiation through the activation of circuitry in the brainstem, including the NUCB2/nesfatin-1 neurons [25]. On the other hand, metformin has a beneficial effect on lipid metabolism [11] through the activation of AMPK, the lowering of the level of serum triglyceride and the elevation of the HDL-cholesterol concentration. In fact, a previous study showed the direct enhancement of fat oxidation in skeletal myotubes and adipocytes in response to metformin treatment *in vitro* [13, 26]. Furthermore, metformin is related to adaptive thermogenesis such as diet-induced thermogenesis [19, 20], which plays an important role in the regulation of body weight and body composition [27]. However, although it’s quite promising that the administration of metformin bring about a reduction of fat amount by a facilitation of fat oxidation, there has been no report so far demonstrating metformin’s effect on fat oxidation together with its effect on body fat reduction *in vivo*. In this context, we tried to clarify the beneficial effects of metformin on body weight, which have been investigated and discussed directly in regard to fat oxidation *in vivo*.

The administration of metformin significantly suppressed the post-prandial elevation of glucose concentration in both healthy subjects and diabetic patients through the amelioration of insulin resistance or the direct augmentation of anaerobic glycolysis, as indicated by the elevation of plasma lactate concentration. Furthermore, the inhibitory effect of metformin on gluconeogenesis might have been involved which was demonstrated in pyruvate tolerance test in the present study. The suppression of the post-prandial increase of triglyceride levels observed in diabetic patients could largely be attributable to the amelioration of insulin resistance and the activation of lipoprotein lipase[28]. While it is feasible that metformin might directly suppress the production of triglyceride and activate fat oxidation through the activation of AMPK in the liver, the increase of post-prandial RQ in response to metformin treatment probably reflects the enhancement of anaerobic glycolysis and does not support this hypothesis.

On the other hand, metformin was found to suppress RQ significantly in the fasted state in both healthy subjects and diabetic patients. This result is in line with a previous study of obese diabetic patients, which demonstrated that a 16-week period of metformin treatment brought about approximately 3 kg of weight loss, most of which was accounted for by body fat mass–despite the absence of change in EE [5]. In animal studies, it was also demonstrated that RQ in the fasted state significantly decreased without any change in EE in response to metformin treatment, thus confirming the results in human studies. At the time of writing, effects of metformin on energy metabolism, especially on RQ, have rarely been discussed, with the exception of a few reports which demonstrated that there was no change in RQ after metformin treatment in normal weight healthy subjects [29] and diabetic patients [30]. The discrepancy in these results could possibly be attributed to the differences in the methods that were used to measure and evaluate of RQ. Indeed, in the present study, we measured RQ separately during fasted and post-prandial conditions. In contrast, the previous studies measured the average RQ for a whole day. In a whole-day measurement, the increased RQ after meals might cancel the decrease in RQ in response to fasting. The decrease of fasting RQ suggests the dominant utilization of fat as an energy source during this period.

In line with the results of energy metabolism, animal studies showed that the administration of metformin led to a reduction of body fat independent of its anorexigenic effects. Thus, it is feasible that beneficial effects of metformin on body weight can largely be attributed to its effects on fat metabolism and oxidation in this study. Metformin’s effects on fat oxidation coincide naturally with its activation of AMPK in the liver, and the inactivation of ACC by AMPK results in a decrease in malonyl-CoA, higher CPT-1 activity and the enhancement of fatty acid oxidation [18]. In the present study, we clearly demonstrated that metformin significantly enhanced the phosphorylation of AMPK leading to the phosphorylation and suppression of ACC, then increased the levels of fat oxidation-related enzymes such as acyl-CoA synthase, CPT-1 and acyl-CoA dehydrogenase. These results account well for its fat oxidation mechanisms. PDK is a kinase enzyme which inactivates the pyruvate dehydrogenase enzyme through its phosphorylation (using ATP), which leads to the suppression of oxidative glycolysis. Thus, the enhancement of PDK by metformin, which was observed in the present study, confirms a potential shift of energy source from glycolysis to fat oxidation. On the other hand, metformin’s effects on UCP-1 and UCP-3 shown in the present study are in line with previous reports [20, 27], and could well explain the reduction of visceral fat taking an enhancement of diet-induced thermogenesis (DIT) during dark phase. In this aspect, a slight but significant decrease of RQ during dark phase can be reflected by the increase of adaptive thermogenesis in this period. An absorptive RQ decrease is the different result from that in human study in which post-prandial RQ was enhanced by metformin treatment. This discrepant results can be partly explained by the difference of BAT amount, which is abundant in mice and scarce in adult human [31].

This study is associated with several limitations. First, the end of the light phase (19:00) under restricted feeding conditions in which rats were only fed during the dark phase does not correctly coincide with the fasting state in humans because the motility of the gut and the absorption of nutrients are quite different between in these 2 species. These differences may explain the differences between humans and rats in the fasting lactate concentration and post-prandial RQ, as well as the similar enhancement of fat oxidation-related enzyme expressions during both of the states in rats. Second, because it is complicated and labor intensive to directly measure insulin sensitivity, we instead measured the serum insulin concentrations to estimate insulin sensitivity. Third, we measured fat oxidation-related enzyme expressions in the liver instead of in adipose tissue. A previous report, which used a liver perfusion technique in a diet-induced overweight *in vivo* rat model, demonstrated that metformin treatment significantly decreased body weight and suppressed the cumulative triglyceride output from the liver, suggesting a change of hepatic fatty acid metabolism from lipogenesis toward fat oxidation [32]. This finding was in line with our results. Furthermore, with regard to cross-talk between liver and adipose tissue, there are many literatures that reported a decrease of visceral fat and an acceleration of fat oxidation related enzymes in the liver simultaneously [33, 34]. Thus, metformin is capable of accelerating the oxidation of fat in the liver, thereby leading to a decrease of visceral fat or body weight. Finally, the dose of metformin that was used for rats was around 250mg/kg per day, with water consumption taken into consideration. While this dose is similar or somewhat smaller than the dose of previous reports in which metformin was administered to rats through their drinking water [35, 36], it is estimated to be approximately 10 times higher than that in human subjects (about 30mg/kg per day). The concern for lactic acidosis, however, has been swept because of the similar pH levels in arterial blood of rats with or without metformin treatment (data not shown).

In conclusion, it is feasible that the long-term administration of metformin brought about a shift of the fuel source for fat oxidation in both human and animal experiments, and a significant decrease of visceral fat volume was noted in animal experiments. Furthermore, metformin treatment enhanced DIT-related UCPs expressions, which may have partly contributed to the reduction of visceral fat observed in animal experiments. We demonstrated *in vivo* that the administration of metformin may cause visceral fat reduction through a possible mechanism of fat oxidation enhancement that is independent of its appetite suppressive effect.

## Supporting information

S1 Table Primer sequences. (DOCX)
