# Supplementary material for: Beneficial effects of metformin on energy metabolism and visceral fat volume through a possible mechanism of fatty acid oxidation in human subjects and rats
Source: PLoS One. 2017 Feb 3;12(2):e0171293. doi: 10.1371/journal.pone.0171293 (PMC5291441; doi:10.1371/journal.pone.0171293)
Supplement: S1 Table — (DOCX) [file pone.0171293.s001.docx]

S1 Table. Primer sequences

*Cpt1*

forward 5’-CTTTGGTACAGGGCTCTGGG -3’

reverse 5’-TACAACATGGGCTTCCGACC -3’ (product length; 168 bp)

*Acs*

forward 5’-CATTTGCAGCCAAGATCGGG -3’

reverse 5’-TCAGTGTCGGTGTCAGAAGC -3’ (product length 195 bp)

*Pdk*

forward 5’-AAAACCGCCCTTTCCTGACA -3’

reverse 5’-AGGGGCATTCCGTGAATTGT -3’ (product length; 168bp)

*Acad*

forward 5’-TCCGCTTCCATGGCGAAATA -3’

reverse 5’-GTACCACCGTAGATCGGCTG -3’ (product length; 152 bp)

*Ucp1*

forward 5’-CCTCTCCGGTGGATGTGGTAAA -3’

reverse 5’-CGCAGAAAAGAAGGCGCAAA -3’ (product length; 213 bp)

*Ucp3*

forward 5’-TACAGAACCATCGCCAGGGA -3’

reverse 5’-TATCGGGTCTTTACCACATCCA -3’ (product length; 142 bp)

*Gapdh*

forward 5’-GTCTTCACCACCATGGAGAAGG -3’

reverse 5’-TCATGGATGACCTTGGCCAG -3’ (product length; 197 bp)
